# The transmembrane domain of *N* –acetylglucosaminyltransferase I is the key determinant for its Golgi subcompartmentation

**DOI:** 10.1111/tpj.12671

**Published:** 2014-09-17

**Authors:** Jennifer Schoberer, Eva Liebminger, Ulrike Vavra, Christiane Veit, Alexandra Castilho, Martina Dicker, Daniel Maresch, Friedrich Altmann, Chris Hawes, Stanley W Botchway, Richard Strasser

**Affiliations:** 1 Department of Applied Genetics and Cell Biology, University of Natural Resources and Life SciencesMuthgasse 18, Vienna, 1190, Austria; 2 Department of Chemistry, University of Natural Resources and Life SciencesMuthgasse 18, Vienna, 1190, Austria; 3 Department of Biological and Medical Sciences, Faculty of Health and Life Sciences, Oxford Brookes UniversityHeadington, Oxford, OX3 0BP, UK; 4 Research Complex at Harwell, Central Laser Facility, Science and Technology Facilities Council, Rutherford Appleton LaboratoryHarwell-Oxford, Didcot, OX11 0QX, UK

**Keywords:** *Arabidopsis thaliana*, glycosyltransferase, Golgi apparatus, *N*-glycan processing, *Nicotiana benthamiana*, protein–protein interaction, transmembrane domain, type-II membrane protein

## Abstract

Golgi-resident type–II membrane proteins are asymmetrically distributed across the Golgi stack. The intrinsic features of the protein that determine its subcompartment-specific concentration are still largely unknown. Here, we used a series of chimeric proteins to investigate the contribution of the cytoplasmic, transmembrane and stem region of *Nicotiana benthamiana N*–acetylglucosaminyltransferase I (GnTI) for its *cis*/medial-Golgi localization and for protein–protein interaction in the Golgi. The individual GnTI protein domains were replaced with those from the well-known *trans*-Golgi enzyme α2,6–sialyltransferase (ST) and transiently expressed in *Nicotiana benthamiana*. Using co-localization analysis and *N*–glycan profiling, we show that the transmembrane domain of GnTI is the major determinant for its *cis*/medial-Golgi localization. By contrast, the stem region of GnTI contributes predominately to homomeric and heteromeric protein complex formation. Importantly, in transgenic *Arabidopsis thaliana*, a chimeric GnTI variant with altered sub-Golgi localization was not able to complement the GnTI-dependent glycosylation defect. Our results suggest that sequence-specific features in the transmembrane domain of GnTI account for its steady-state distribution in the *cis*/medial-Golgi in plants, which is a prerequisite for efficient *N*–glycan processing *in vivo*.

## Introduction

The Golgi apparatus is the central biosynthetic organelle of the secretory pathway. It receives cargo proteins, polysaccharides and lipids from the endoplasmic reticulum (ER), subjects them to extensive processing in different subcompartments and transports the cargo to other destinations within the endomembrane system. The compartmentation of biosynthetic activities in different cisternae of a polarized Golgi stack is a major function of the Golgi. Many processing steps involve modifications of protein- or lipid-bound oligosaccharides that are carried out by a large number of Golgi-resident glycosyltransferases and glycosidases. The overlapping but non-uniform distribution of these glycosylation enzymes across the Golgi stack is well documented, and has been shown for plants by immunoelectron and confocal microscopy (Saint-Jore-Dupas *et al.*, 2006; Reichardt *et al.*, 2007; Chevalier *et al.*, 2010; Schoberer *et al.*, 2010). The specialized Golgi architecture provides an excellent means to asymmetrically distribute these enzymes and consequently ensures the sequential order of glycan processing on transiting cargo. The concentration of Golgi glycosylation enzymes in distinct Golgi domains is a prerequisite for controlled glycan biosynthesis, as different glycosyltransferases and glycosidases may compete for identical substrates and certain reaction products inhibit the action of other enzymes resulting in partially processed glycans. The removal of mannose residues from hybrid *N*–glycans by Golgi-α–mannosidase II, for example, is blocked by prior action of β1,4–galactosyltransferase (Palacpac *et al.*, 1999; Bakker *et al.*, 2001). Yet, despite our understanding of the functional importance of compartmentation of Golgi glycosylation enzymes, the signals and underlying mechanisms required to establish and maintain the asymmetric distribution are still largely unknown in plants and other organisms (Tu and Banfield, 2010; Schoberer and Strasser, 2011; Oikawa *et al.*, 2013).

The dynamic distribution and trafficking of resident proteins in the Golgi is dependent on the overall cargo transport mechanism through this organelle, which remains controversial. The constant flux of cargo and the dynamic distribution of Golgi-integral membrane proteins suggest that the polar distribution of Golgi-resident proteins is achieved by a coordinated interplay of retrieval and retention mechanisms. The majority of the Golgi glycosyltransferases and glycosidases are type–II membrane proteins consisting of a short cytoplasmic tail, a single transmembrane region, a flexible stem and a large catalytic domain that faces the lumen of the Golgi cisternae (Schoberer and Strasser, 2011). This basic domain organisation is similar between Golgi enzymes from different eukaryotic kingdoms and the underlying Golgi-targeting/retention or retrieval mechanisms also seem to be highly conserved between species (Boevink *et al.*, 1998; Wee *et al.*, 1998; Palacpac *et al.*, 1999; Bakker *et al.*, 2001). Consequently, these enzymes should contain either a specific amino acid sequence motif or a protein conformation that leads to the observed steady-state localization within the Golgi apparatus. Different mechanisms have been proposed for Golgi-localization and the sorting of glycosylation enzymes. The oligomerization or kin recognition model is based on the possibility that glycosylation enzymes can form homo- or heteromeric protein complexes that are together retained or concentrated in distinct regions of the Golgi apparatus (Machamer, 1991; Nilsson *et al.*, 1994). For *N*–glycan processing enzymes distinct Golgi protein complex formation has been described in mammalian cells as well as in plants (Hassinen *et al.*, 2010; Schoberer *et al.*, 2013). By contrast, the bilayer thickness model postulates that changes in the thickness of the lipid bilayer restrict the forward transport of proteins with shorter transmembrane domains, and thus could play an important role in the retention of proteins in different Golgi cisternae (Bretscher and Munro, 1993). Experimental evidence revealed that the transmembrane domain length and/or sequence composition are important determinants of subcellular distribution in different eukaryotes (Munro, 1995; Brandizzi *et al.*, 2002a; Saint-Jore-Dupas *et al.*, 2006). These studies mainly examined the contribution of the transmembrane domain length in Golgi retention, in comparison with other organelles such as the ER and plasma membrane, but the impact on sub-Golgi localization has not been addressed in detail.

In addition, recent studies from yeast have highlighted a role of the short cytoplasmic tail of glycosylation enzymes as a determinant of Golgi-retention and intra-Golgi trafficking (Schmitz *et al.*, 2008; Tu *et al.*, 2008). In this receptor-mediated retrieval model the coat protein complex–I (COPI) binds, via the peripheral membrane protein Vps74p, to a specific amino acid sequence stretch in the cytoplasmic tail of glycosyltransferases, leading to Golgi retention or retrograde trafficking. In plants, the interaction of the cytoplasmic tail of the multi-pass transmembrane protein EMP12 with COPI maintains its Golgi retention (Gao *et al.*, 2012), but the impact of the cytoplasmic tail on Golgi-localization of type–II membrane proteins is unclear.

Here, we addressed the question of whether such mechanisms involving either the cytoplasmic tail or the transmembrane domain or the stem region are responsible for the steady-state Golgi distribution of *N. benthamiana N*–acetylglucosaminyltransferase I (GnTI). GnTI is a *cis*/medial-Golgi-resident type–II membrane protein that plays a key role in *N*–glycan processing, because it initiates the formation of complex *N*–glycans in animals and plants (Burke *et al.*, 1994; Schoberer *et al.*, 2009). We focused on the N–terminal cytoplasmic transmembrane and stem (CTS) region of GnTI, which is sufficient for sub-Golgi localization in leaves of *Nicotiana benthamiana* plants, and performed domain-swap experiments. The cytoplasmic tail, transmembrane or stem region of GnTI was exchanged with the corresponding regions from rat α–2,6–sialyltransferase (ST), which is the most widely used *trans*-Golgi marker in plants (Boevink *et al.*, 1998). We examined the contribution of the individual protein regions to their subcellular localization, protein complex formation and ability to restore *N*–glycan processing in the *Arabidopsis thaliana gntI* mutant. Our data provide insights for the specific role of individual GnTI domains in Golgi localization and subsequent *in vivo* function in plants.

## Results

### *N–*Glycan analysis demonstrates differences in the subcellular localization of chimeric type–II membrane proteins

**Figure 1:**
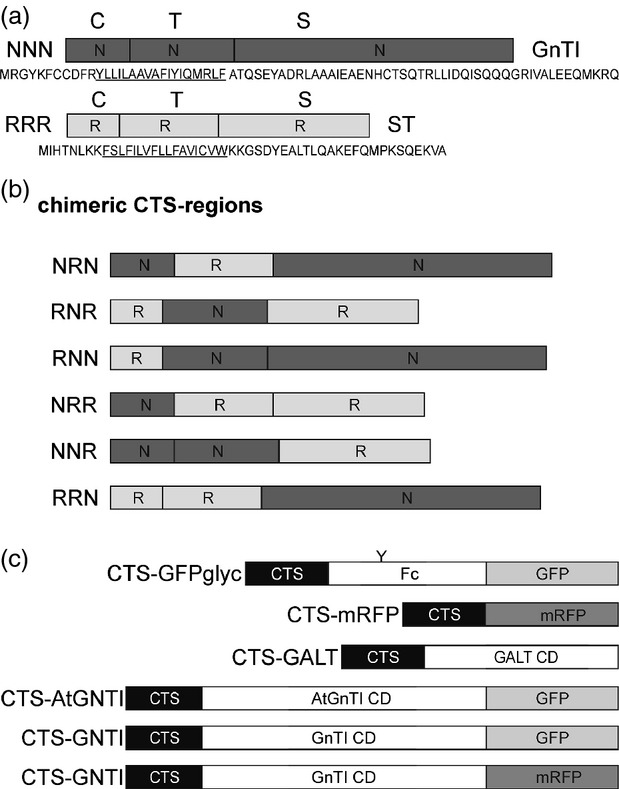
Schematic presentation of protein fusions.(a) The cytoplasmic transmembrane and stem (CTS) regions of *N–*acetylglucosaminyltransferase I (GnTI, NNN) and α–2,6-sialyltransferase (ST, RRR), and corresponding amino acid sequences, are shown. C denotes the short N–terminal cytoplasmic tail; T indicates the transmembrane domain (underlined in the corresponding amino acid sequence) and S depicts the stem region. Domains marked by ‘N’ are from *Nicotiana tabacum* GnTI and ‘R’ indicates domains from ST.(b) Schematic presentation of the chimeric CTS regions derived by exchange of C, T or S regions (NRN, RNR, RNN, NRR, NNR, RRN).(c) Schematic presentation of reporter protein domains that were fused to the CTS regions. ‘Y’ denotes the single *N–*glycosylation site present in GFP_glyc_. The conserved Fc domain from human IgG1 is used for affinity purification. GALT CD harbors the catalytic domain (CD) of human β–1,4-galactosyltransferase. AtGnTI CD harbors the catalytic domain of *Arabidopsis thaliana* GnTI. This construct is expressed under the endogenous *GnTI* promoter from *A. thaliana*. GnTI CD harbors the catalytic domain of *N. tabacum* GnTI.

**Figure 2:**
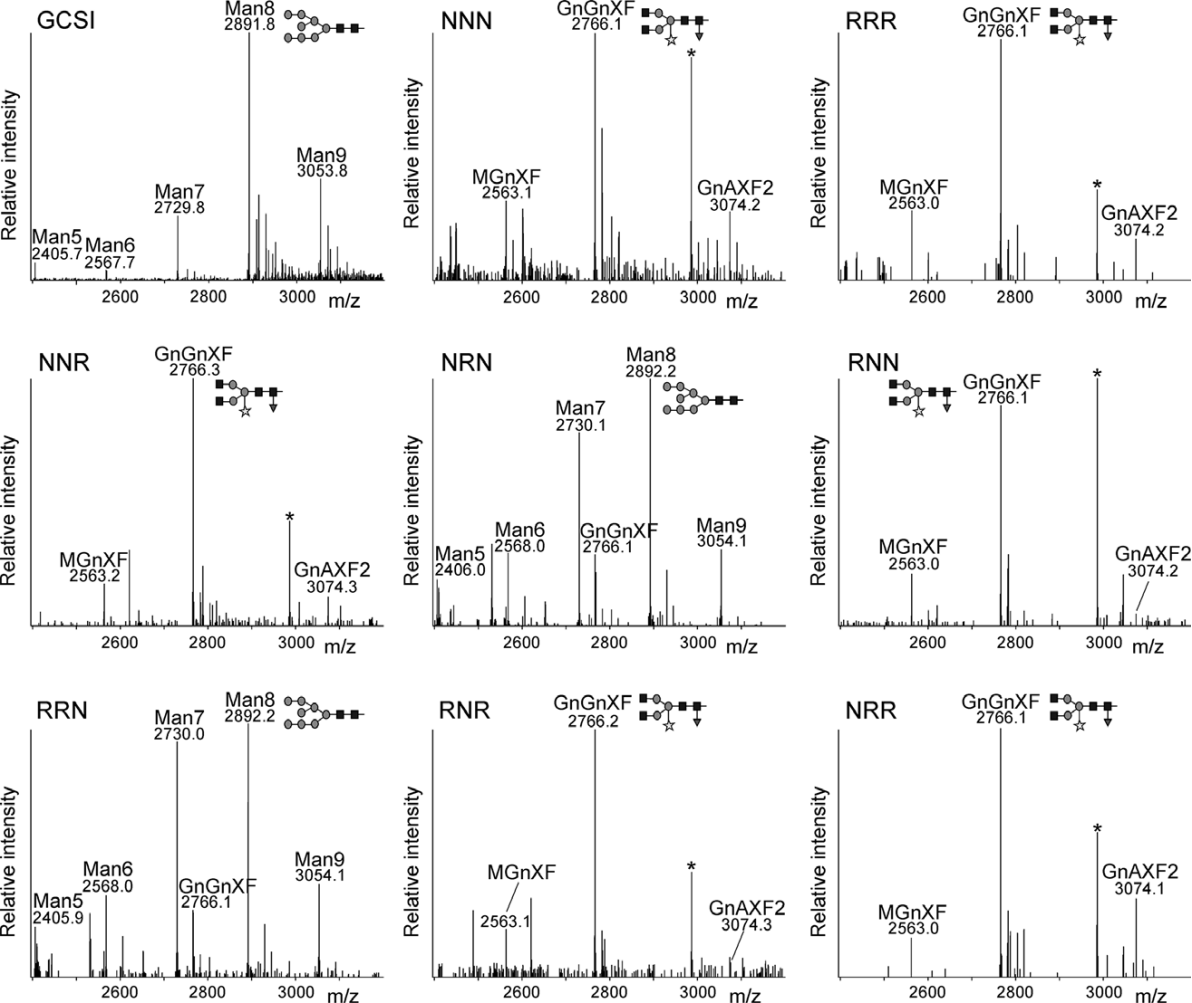
LC-ESI-MS analysis of glycoreporter fusion proteins reveals differences in subcellular localization. Mass spectra of glycopeptides 1 (EEQYNSTYR) derived from the glycoprotein part of GFP_glyc_. GCSI, chimeric construct containing the CTS region from the ER-resident *Arabidopsis thaliana* α–glucosidase I fused to the glycoreporter. Man5 (Man_5_GlcNAc_2_) to Man9 (Man_9_GlcNAc_2_), oligomannosidic *N–*glycans, indicative of ER retention; GnGnXF (GlcNAc_2_XylFucMan_3_GlcNAc_2_), MGnXF (GlcNAcXylFucMan_3_GlcNAc_2_), GnAXF2 GalGlcNAc_2_XylFuc_2_Man_3_GlcNAc_2_) complex *N–*glycans, processed in the Golgi apparatus. The schematic presentation corresponding to the major *N–*glycan peak is given. The asterisk denotes the presence of an unspecific peak.

To examine the role of the N–terminal region in the sub-Golgi localization of *N. benthamiana* GnTI, we generated reporter constructs consisting of chimeric CTS regions from GnTI (NNN) and ST (RRR). We chose the Golgi targeting domains (Figure1a) from these two glycosyltransferases because they lead to an overlapping, but distinct, sub-Golgi distribution when transiently expressed in leaves of *N. benthamiana*, and their CTS regions do not physically interact (Schoberer *et al.*, 2010, 2013). Six chimeric proteins were designed by exchanging the respective cytoplasmic tail, transmembrane domain and luminal stem region (Figure1b). In our first approach, we fused all six chimeric CTS regions to a glycosylation reporter (GFP_glyc_) consisting of the IgG1 heavy chain fragment (Fc domain) and GFP (Figure1c; Schoberer *et al.*, 2009). The Fc domain is used for affinity purification of expressed proteins, and contains a single *N*–glycosylation site that can be used to monitor differences in *N*–glycan processing. To analyze whether the different chimeric CTS regions lead to differences in subcellular localization, the CTS-GFP_glyc_ variants were transiently expressed in leaves of *N. benthamiana*. Purified CTS-GFP_glyc_ proteins were trypsin-digested and peptides were subjected to MS analysis. The *N*–glycosylation profile of NNN-GFP_glyc_ and RRR-GFP_glyc_ displayed almost exclusively a peak corresponding to the complex *N*–glycan GlcNAc_2_XylFucMan_3_GlcNAc_2_ (GnGnXF), which confirms processing in the Golgi apparatus. Peaks representing incompletely processed or further elongated *N*–glycan structures were only found in low quantities (Figure2). The GnGnXF *N*–glycan was also detected as the predominant structure in NNR-GFP_glyc_, RNR-GFP_glyc_, RNN-GFP_glyc_, and NRR-GFP_glyc_. By contrast, the *N*–glycan analysis of NRN-GFP_glyc_ and RRN-GFP_glyc_ revealed primarily peaks corresponding to oligomannosidic *N*–glycans (Man_5_GlcNAc_2_–Man_9_GlcNAc_2_; Figure2) and a peak corresponding to the unglycosylated peptide (Figure S1). The oligomannosidic structures are indicative of retention in the ER, and a similar profile was also detected for the ER-retained reporter GCSI-GFP_glyc_.

### *In vivo* protein galactosylation reveals differences in the Golgi subcompartmentation of chimeric CTS region-containing proteins

**Figure 3:**
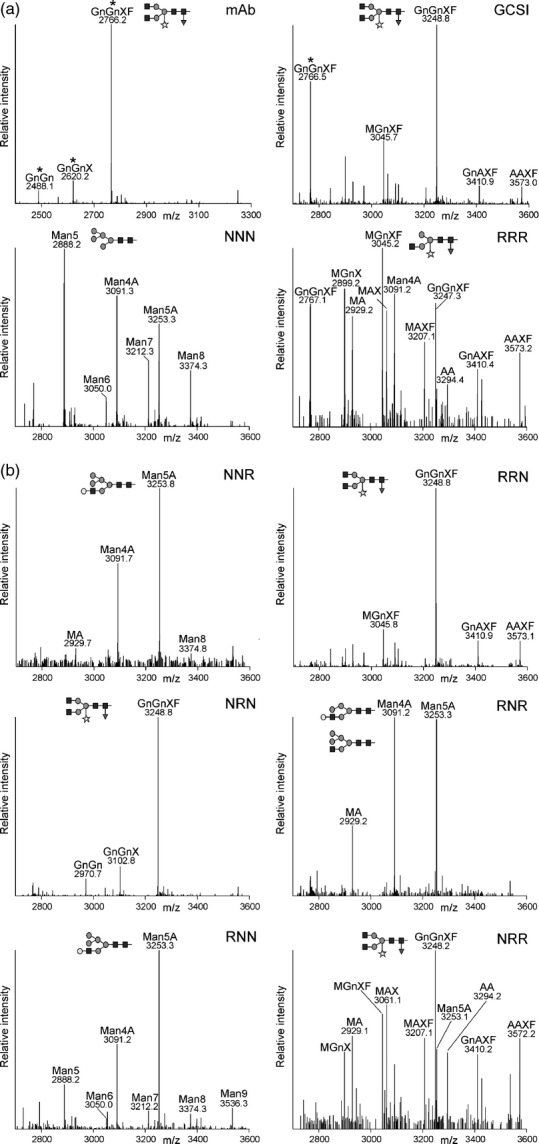
Co-expression of chimeric-GALT and *N–*glycan analysis of a glycoprotein reveal distinct sub-Golgi-targeting regions. LC-ESI-MS of a monoclonal antibody (mAb) co-expressed with chimeric cytoplasmic transmembrane and stem (CTS) regions fused to GALT. Mass spectra of glycopeptides 1 (EEQYNSTYR) or 2 (TKPREEQYNSTYR) derived from the Fc region of the mAb are shown. The two glycopeptides differ by 482 Da and different ratios of the two glycopeptides are generated during the sample preparation by incomplete digestion with trypsin (Stadlmann *et al.*, 2008). Peaks derived from glycopeptides 1 are marked by asterisks.(a) *N–*glycan profiles derived by co-expression of NNN-GALT or RRR-GALT. mAb indicates the *N–*glycan profile in the absence of any GALT enzyme and GCSI shows the profile generated by co-expression of the ER-retained GCSI-GALT. The schematic presentation corresponding to the major *N–*glycan peak is given.(b) *N–*glycan profiles derived by co-expression of mAb with chimeric CTS-GALT enzymes.

Data from previous studies suggest that the attachment of β1,4-linked galactose to *N*–glycans in the Golgi can be used to monitor differences in sub-Golgi localization (Palacpac *et al.*, 1999; Bakker *et al.*, 2001, 2006; Strasser *et al.*, 2009). N–Glycans with β–1,4-linked galactose residues are not normally present in plants, and the responsible β–1,4-galactosyltransferase (GALT) competes with other *N*–glycan processing enzymes for the acceptor substrates. As a consequence of β–1,4-galactosylation, the access of endogenous Golgi-resident enzymes like Golgi-α-mannosidase II (GMII) to their substrates is blocked, resulting in the formation of incompletely processed *N*–glycans (Figure S2). We hypothesized that the co-expression of chimeric CTS-GALT enzymes leads to alterations in *N*–glycosylation dependent on the sub-Golgi distribution of CTS-GALT. To test our approach we fused the CTS regions from GnTI and ST to the catalytic domain of *Homo sapiens* GALT, and analyzed the generated *N*–glycans of a co-expressed monoclonal antibody (mAb), which served as a glycoprotein reporter (Strasser *et al.*, 2009). The mAb *N*–glycan profile obtained by fusion of GALT to the CTS region of the *cis*/medial-Golgi enzyme GnTI unambiguously differed from that derived by RRR-GALT (Figure3a). The glycopeptide profile obtained by co-expression of NNN-GALT consisted mainly of incompletely processed structures (Man5, Man4A/Man5Gn, and Man5A). By contrast, these structures were less abundant when the *trans*-Golgi targeting region RRR was fused to GALT. In that case, complex galactosylated *N*–glycan structures containing xylose and fucose residues (e.g. MAXF, GnAXF and AAXF) were more abundant (Figure3a).

Next, we fused the chimeric CTS regions to the catalytic domain of GALT and co-expressed them with the glycoprotein reporter. The *N*–glycans co-expressed with RNR-, RNN- and NNR-GALT displayed primarily incompletely processed *N*–glycans, being indicative of *cis*/medial-Golgi localization (Figure3b). NRR-GALT generated more fully galactosylated complex *N*–glycans, and thus resembles *trans*-Golgi targeting. Consistent with the previously detected ER retention (Figure2), NRN-GALT and RRN-GALT did not produce significant quantities of galactosylated *N*–glycans, and the *N–*glycan profile was comparable with that from mAb without any co-expressed GALT or from the ER-retained version GCSI-GALT (Figure3a,b). Collectively, these data strongly indicate that the chimeric RNR, RNN and NNR CTS regions concentrate proteins mainly in the *cis*/medial-Golgi, whereas NRR mediates predominately *trans*-Golgi accumulation.

### The transmembrane domain of GnTI plays an important role for its sub-Golgi localization

**Figure 4:**
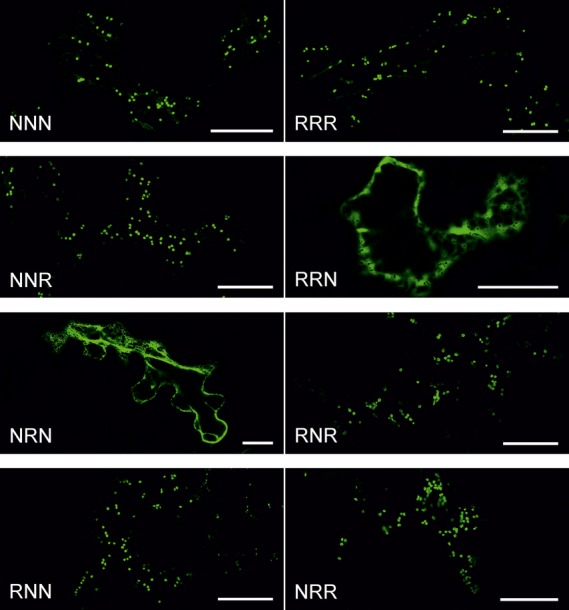
Subcellular localization of fluorescent domain-swap constructs. GFP_glyc_-fused proteins were expressed transiently in *Nicotiana benthamiana* leaf epidermal cells and analyzed by confocal microscopy 3 days post infiltration (dpi). Each confocal image depicts a representative cell expressing the stated GFP_glyc_-fusion (green). Scale bars: 25 μm.

To further investigate the contribution of the individual domains to sub-Golgi localization, we analyzed the subcellular localization of chimeric CTS-GFP_glyc_ variants by live-cell confocal microscopy. As expected, NNR-, RNR-, RNN- and NRR-GFP_glyc_ marked the Golgi (Figure4). In agreement with our data from *N*–glycan analysis, NRN-GFP_glyc_ displayed mainly ER labelling and RRN-GFP_glyc_ showed ER localization, as well as targeting to other subcellular compartments like the cytoplasm (Figure4).

**Figure 5:**
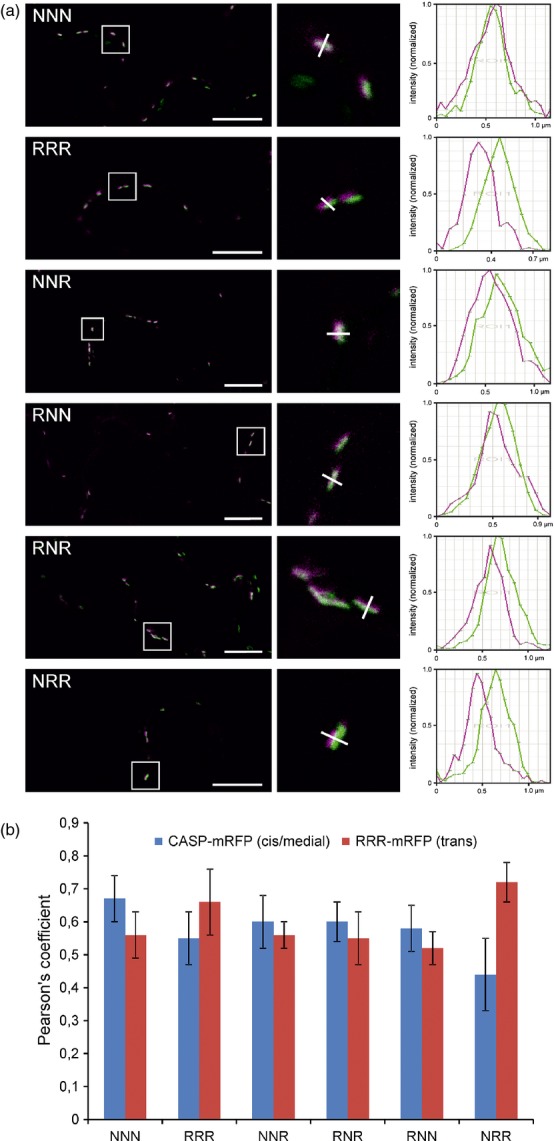
Co-localization analysis shows changes in intra-Golgi localization of fluorescent domain-swap constructs. Fluorescent protein fusions were transiently expressed in *Nicotiana benthamiana* leaf epidermal cells and analyzed by live-cell confocal microscopy (3 dpi) without fixation or inhibition of Golgi stack motility. Confocal images produced in (a) were used for co-localization analyses in (b).(a) Merged confocal images in the left panel show representative cells co-expressing GFP_glyc_-fused proteins (green) with the reference marker mRFP-AtCASP (magenta), an *Arabidopsis cis*/medial-Golgi matrix protein, in Golgi stacks of live cells. Co-localization appears in white. The boxed areas are shown as magnifications in the middle panel. The white line drawn across representative Golgi stacks was used to generate fluorescence intensity profiles shown in the right panel, which reflect the distribution of the fluorescence intensity of the respective GFP fusion (green) and mRFP-AtCASP (magenta) along the line. Scale bars: 10 μm.(b) Co-localization analyses of GFP_glyc_-fused proteins co-expressed with the *cis*/medial-Golgi marker mRFP-AtCASP and the non-plant *trans*-Golgi marker RRR-mRFP, respectively, using the Pearson's correlation coefficient.

Next, we used confocal microscopy to determine the sub-Golgi distribution of the chimeric CTS-GFP_glyc_ proteins in comparison with the *cis*/medial-Golgi located Golgi matrix protein AtCASP-mRFP (Renna *et al.*, 2005; Osterrieder *et al.*, 2009; Schoberer *et al.*, 2010). The fluorescence profiles for chimeric CTS-GFP_glyc_ and AtCASP-mRFP across Golgi stacks revealed clear differences for NRR, whereas NNR, RNN and RNR shifted to a lesser extent (Figure5a). To more precisely analyze the sub-Golgi localization, we calculated the Pearson's correlation coefficient for co-localization with AtCASP-mRFP and the *trans*-Golgi marker RRR-mRFP. Although the correlation between NRR-GFP_glyc_ and AtCASP-mRFP was substantially lower than for NNN-GFP_glyc_ and AtCASP-mRFP, the NNR, RNR and RNN correlation was more similar to NNN (Figures5b and S3). By contrast, NRR-GFP_glyc_ displayed a strong correlation with RRR-mRFP. On the other hand, like NNN, the NNR, RNR and RNN regions displayed a significantly lower correlation. Consistent with the *N–*glycan analysis, these data highlight that the transmembrane domain plays an important role for *cis/*medial-Golgi localization of GnTI, whereas the cytoplasmic tail and stem region are not involved in sub-Golgi distribution.

### The stem region of GnTI is relevant for homo- and heterodimer formation

**Figure 6:**
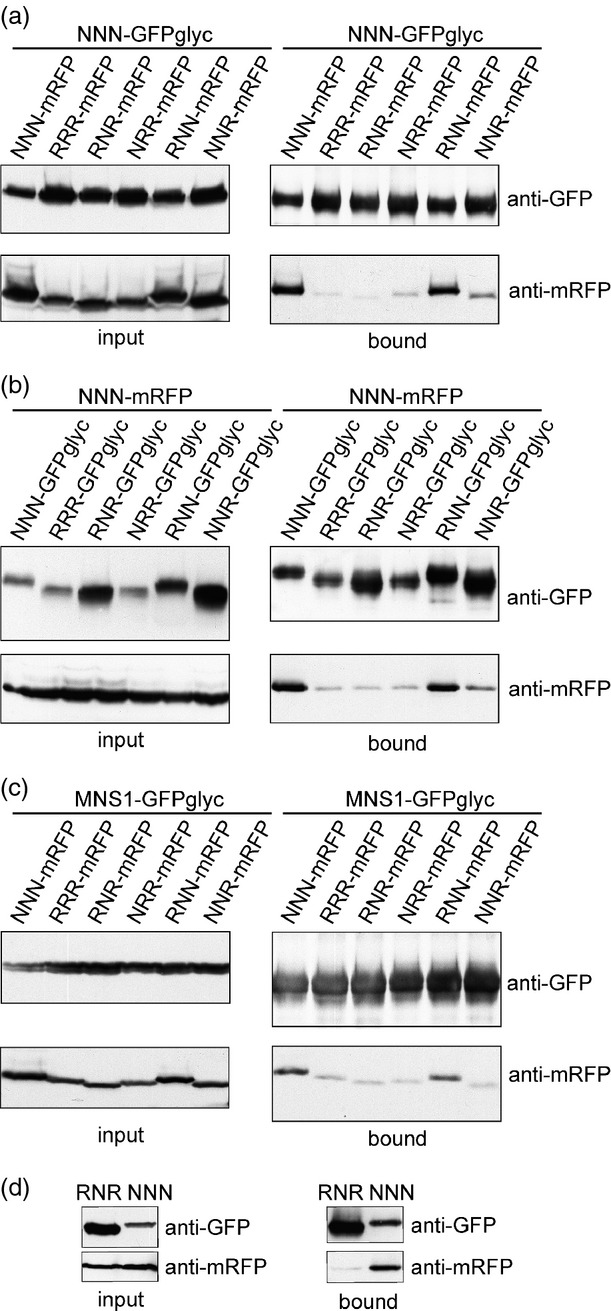
The stem region of GnTI is mainly responsible for protein–protein interactions. The indicated proteins were transiently co-expressed in *Nicotiana benthamiana* leaves and the GFP-tagged proteins were purified by incubation with protein A (a–c) or GFP-coupled beads (d). Immunoblot analysis of protein extracts (input = before incubation with beads) and eluted samples (bound = fraction eluted from beads) with anti-GFP and anti-mRFP antibodies.(a) NNN-GFP_glyc_ was precipitated and co-purified chimeric CTS-mRFP was monitored by immunoblotting.(b) Chimeric CTS-GFP_glyc_ was precipitated and co-purified NNN-mRFP was monitored by immunoblotting.(c) MNS1-GFP_glyc_ was precipitated and co-purified chimeric CTS-mRFP was monitored by immunoblotting.(d) RNR-GNTI-GFP and NNN-GNTI-GFP were purified by binding to GFP-coupled beads and co-purified NNN-GNTI-mRFP was analyzed by immunoblotting.

In a previous study, we have demonstrated that *N. benthamiana* GnTI forms homodimers in the Golgi apparatus, which is mediated by the N–terminal CTS region (Schoberer *et al.*, 2013). To test the contribution of the different domains to protein–protein interactions, we co-expressed NNN-GFP_glyc_ with mRFP-tagged chimeric CTS regions (RNR, NRR, RNN and NNR) in *N. benthamiana* leaves and purified GnTI-GFP_glyc_ by binding to protein A. Immunoblot analysis revealed that the quantity of co-purified RNN-mRFP was similar to NNN-mRFP, whereas binding of NNR-mRFP, RNR-mRFP and NRR-mRFP was as low as RRR-mRFP (Figure6a), which does not interact with GnTI-GFP_glyc_ (Schoberer *et al.*, 2013). Similarly, when NNN-mRFP was co-expressed with chimeric CTS-GFP_glyc_ interaction was only found for RNN (Figure6b). In addition, when MNS1-GFP_glyc_, which forms a heteromeric complex with GnTI (Schoberer *et al.*, 2013), was used to co-purify chimeric CTS-mRFP proteins, considerable quantities of the heteromeric MNS1/RNN complex were detected (Figure6c).

To examine whether the catalytic domain plays any role in complex formation, we fused the chimeric RNR region to the full-length catalytic domain of *N. benthamiana* GnTI (RNR-GNTI-GFP), co-expressed RNR-GNTI-GFP with the control NNN-GNTI-mRFP (GnTI CTS region fused to the catalytic domain) and performed co-immunoprecipitation (co–IP) followed by immunoblot detection. In agreement with our previous data, no marked interaction could be found between RNR-GNTI-GFP and NNN-GNTI-mRFP (Figure6d). Collectively, the co–IP experiments suggest that the GnTI stem region is primarily required for complex formation.

To verify the co–IP results and test for direct interaction of the individual domains, we selected specific chimeric CTS-mRFP fusions and tested the *in vivo* GnTI interactions using two-photon excitation fluorescence resonance energy transfer- fluorescence lifetime imaging (FRET-FLIM; Schoberer *et al.*, 2013). The average excited-state fluorescence lifetime of the NNN-GFP_glyc_ donor was 2.44 ns in the absence of an acceptor fluorophore (Table1). The presence of co-expressed NNN-mRFP led to a significant quenching of the donor lifetime to an average of 2.08 ns (14.56% FRET efficiency), which is indicative of a strong protein–protein interaction. Similarly, co-expression with RNN-mRFP produced FRET efficiency values of 10.79%, indicative of physical interaction and dimer formation in the Golgi membrane. By contrast, donor quenching was less efficient in the presence of RNR-mRFP, indicating no or only a weak interaction, and the values obtained in the presence of NRR-mRFP were in the range of those for RRR-mRFP, which does not physically interact with GnTI (Schoberer *et al.*, 2013). Taken together, the FRET-FLIM data are consistent with the co–IP results and highlight the importance of the stem region in GnTI homodimer formation.

**Table 1:** Fluorescence resonance energy transfer (FRET) efficiency determined by fluorescence lifetime imaging (FLIM)

Donor	Acceptor	*τ*_*D*_ ± SD (ns)	*τ*_DA_ ± SD (ns)	Δ*τ* (ns)	*E* (%)
NNN-GFP_glyc_	NNN-mRFP	2.44 ± 0.06 (*n* = 412)	2.08 ± 0.09 (*n* = 204)	**0.36**	**14.56**
NNN-GFP_glyc_	RRR-mRFP	2.44 ± 0.06 (*n* = 412)	2.36 ± 0.06 (*n* = 238)	0.08	3.35
NNN-GFP_glyc_	RNR-mRFP	2.44 ± 0.06 (*n* = 412)	2.26 ± 0.05 (*n* = 217)	0.18	7.34
NNN-GFP_glyc_	RNN-mRFP	2.44 ± 0.06 (*n* = 412)	2.18 ± 0.07 (*n* = 185)	**0.26**	**10.79**
NNN-GFP_glyc_	NRR-mRFP	2.44 ± 0.06 (*n* = 241)	2.36 ± 0.06 (*n* = 158)	0.08	3.09

*τ*_*D*_, lifetime of the donor in the absence of the acceptor; *τ*_DA_, lifetime of the donor in the presence of the acceptor; Δ*τ*, lifetime contrast (*τ*_*D*_ − *τ*_DA_); *E*, FRET efficiency; ns, nanosecond; SD, standard deviation. A minimum decrease in the average excited-state fluorescence lifetime of the donor molecule by 0.20 ns, or 8%, in the presence of the acceptor molecule was considered to be indicative of an interaction. Protein pairs and respective values indicating an interaction are shown in bold.

### Complementation of the *N*–glycan processing defect requires correct sub-Golgi targeting signals

**Figure 7:**
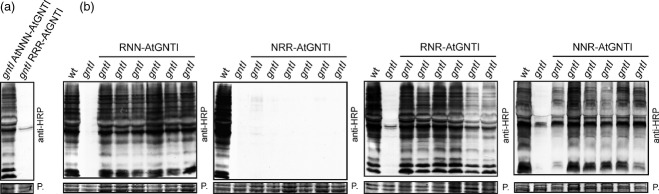
Complementation of the *Arabidopsis thaliana gntI* mutant by expression of chimeric CTS regions fused to the catalytic domain of *A. thaliana* GnTI. Proteins were extracted from 5–week-old soil-grown plants, separated by SDS-PAGE and complex *N–*glycans were detected by immunoblotting using antibodies directed against β–1,2-xylose and α–1,3-fucose containing complex *N–*glycans.(a) Protein extracts from *gntI* expressing AtNNN-AtGNTI or RRR-AtGNTI.(b) Protein extracts from *gntI* expressing RNN-AtGNTI, NRR-AtGNTI, RNR-AtGNTI or NNR-AtGNTI. Ponceau S (P.) staining serves as the loading control.

Next, to examine whether the findings obtained from the chimeric reporter proteins can be applied to full-length GnTI and its *in vivo N–*glycan processing activity, we generated transgenic *A. thaliana gntI* plants expressing the chimeric CTS regions fused to the catalytic domain of *A. thaliana* GnTI (AtGNTI). To exclude any overexpression effect the chimeric AtGNTI proteins were expressed under the control of the endogenous *GnTI* promoter. The complementation of the *N–*glycan processing defect of *gntI* plants was analyzed by the immunoblotting of protein extracts with antibodies directed against complex *N–*glycans. As expected, AtNNN-AtGNTI complemented the *N–*glycan processing defect of *gntI*, and restored complex *N–*glycan formation (Figure7a). By contrast, RRR-AtGNTI expression did not rescue the *N–*glycan processing defect, suggesting that the ST-mediated *trans*-Golgi targeting of GnTI is not functional. Consistent with an altered steady-state sub-Golgi distribution, NRR-AtGNTI-expressing *gntI* plants did not produce complex *N–*glycans (Figure7b). On the other hand, RNN-AtGNTI, RNR-AtGNTI and NNR-AtGNTI were functional, and rescued the complex *N–*glycan processing defect. In summary, our data indicate that distinct domains within the CTS region are crucial for the sub-Golgi localization, and subsequently for the *in vivo* function of GnTI, in plants.

## Discussion

A central biosynthetic function of the Golgi is the modification of protein- and lipid-bound glycans and polysaccharides. Typically, this function is carried out by type–II membrane proteins that are asymmetrically distributed in some kind of assembly line across the Golgi stack. In yeast and mammalian cells, different protein regions have been found to contribute to the Golgi localization of glycan-modifying enzymes (Grabenhorst and Conradt, 1999; Fenteany and Colley, 2005; Schmitz *et al.*, 2008; Tu *et al.*, 2008). In contrast, the sub-Golgi targeting determinants of most glycosyltransferases and glycosidases are largely unknown. Dependent on the mode of cargo transport through the Golgi these domains contain either retention signals (vesicular transport model) or retrograde trafficking signals (cisternal maturation model) (Rabouille and Klumperman, 2005). Here, we tested the contribution of the different domains from the N–terminal Golgi-targeting region of the *cis*/medial-Golgi enzyme GnTI for sub-Golgi localization. To obtain detailed information on sub-Golgi targeting, we used live-cell imaging (Figures4 and 5) and took advantage of sensitive biochemical approaches based on the monitoring of changes in *N–*glycan processing (Figures2 and 3). Analysis of the *N–*glycan profile of a chimeric glycoprotein (Figure2) provided information on the topology of the expressed proteins, as only correctly orientated forms are glycosylated in the lumen of the ER, and this allowed us to discriminate between ER retention and Golgi targeting. Interestingly, the *N–*glycan modifications appeared independent of the *cis*/medial- or *trans*-Golgi targeting regions, indicating that the dynamic distribution of *N–*glycan processing enzymes leads to contact between cargo and processing enzymes from other Golgi cisternae; however, because of substrate competition the impact on sub-Golgi compartmentation was clearly discernible when chimeric CTS regions were fused to the catalytic domain of GALT (Figure3).

Strikingly, most Golgi-resident type–II membrane proteins contain a short tail that faces the cytoplasm. In mammals as well as in plants, the short cytoplasmic region contains a basic amino acid motif that is required for COPII vesicle interaction and ER export (Giraudo and Maccioni, 2003; Schoberer *et al.*, 2009). Earlier studies with mammalian glycosyltransferases showed that these cytoplasmic tails are also implicated in sub-Golgi localization, which could be mediated by binding of cytosolic proteins (Uliana *et al.*, 2006). For rat ST as well as for human GnTI it has been proposed that the cytoplasmic domain contributes to Golgi localization (Burke *et al.*, 1994; Fenteany and Colley, 2005); however, our data from swapping the cytoplasmic tails clearly show that these N–terminal amino acid regions are not involved in *cis*/medial- or *trans*-Golgi concentration of GnTI and ST in plants (Figures3 and 5). Moreover, the GnTI variant with the cytoplasmic tail from ST was fully functional *in vivo* (Figure7). In yeast, the peripheral Golgi protein Vps74p interacts with motifs in the cytoplasmic tails of glycosyltransferases, and subsequently functions as a glycosyltransferase sorting receptor for their retrograde trafficking and/or Golgi retention (Schmitz *et al.*, 2008; Tu *et al.*, 2008). Further studies revealed that GOLPH3, the mammalian Vps74p ortholog, interacts with a conserved amino acid sequence motif present in the cytoplasmic tail of distinct glycosyltransferases (Ali *et al.*, 2012). Interestingly, *A. thaliana* and other plants seem to lack Vps74p/GOLPH3 homologs, and, so far, a conserved sequence motif could not be detected in the cytoplasmic tail of plant type–II membrane proteins (Schoberer and Strasser, 2011), which suggests that there are fundamental differences in the mechanisms that concentrate glycan modifying type–II membrane proteins in plants and in other kingdoms.

Using time-resolved fluorescence imaging we recently detected the formation of homo- and heterodimers between *N–*glycan processing enzymes located in the early Golgi (Schoberer *et al.*, 2013). The organization in multi-protein complexes might contribute to their Golgi localization and/or modulate their activity. It was previously proposed that the oligomerization or kin recognition of glycosylation enzymes in mammals is important for Golgi retention, by excluding large multi-enzyme complexes from vesicles that mediate cargo transport (Machamer, 1991). For human GnTI the formation of homodimers has been described and it has been suggested that oligomerization plays a major role for Golgi retention (Hassinen *et al.*, 2010). In line with data for mammalian GnTI (Nilsson *et al.*, 1996), we observed that the stem region of *N. benthamiana* GnTI is involved in homomeric and heteromeric complex formation (Figure6); however, our data also indicate that the protein–protein interaction is not implicated in sub-Golgi compartmentation. In the absence of a strong interaction, the sub-Golgi localization of Golgi-resident GnTI-chimeras does not appear to be considerably altered, suggesting that the complex formation is not a prerequisite for the *cis*/medial-Golgi concentration of GnTI. Interestingly, the almost full restoration of complex *N–*glycan formation in transgenic *gntI* plants expressing chimeras that display no or weak protein–protein interaction (Figure7) hints that a kin recognition process plays only a minor role for the functionality of GnTI. Nonetheless, it cannot be excluded that homomeric or heteromeric protein complexes are required to modulate or fine-tune the activity of GnTI. Such subtle modifications might be required in certain cell types or under adverse environmental conditions (Kang *et al.*, 2008). Enhanced *in vitro* enzyme activity because of complex formation has, for example, been demonstrated for plant glycosyltransferases involved in arabinogalactan biosynthesis (Dilokpimol *et al.*, 2014). Moreover, there is emerging experimental evidence that complex formation of Golgi-resident proteins occurs quite frequently in plants (Oikawa *et al.*, 2013); however, the biological relevance of these complexes in the modulation of enzyme activities or substrate specificities, and finally in the regulation of glycan biosynthesis, remains to be shown.

### The transmembrane domain is implicated in sub-Golgi localization of *N. benthamiana* GnTI

An important role of the transmembrane domain for Golgi retention was described for mammalian *N–*glycan processing enzymes such as GALT and ST (Nilsson *et al.*, 1991; Munro, 1995). For a chimeric protein containing the transmembrane domain of rabbit GnTI, only partial Golgi retention was described, indicating that several regions cooperatively mediate its Golgi localization (Burke *et al.*, 1994). Our data provide evidence that the transmembrane domain is the key determinant for the sub-Golgi distribution of plant GnTI. The chimeras containing the GnTI transmembrane domain flanked by ST regions were predominately found in the same compartment as GnTI (Figures3 and 5) and, importantly, the RNR-AtGNTI chimeric protein was functional when expressed under native conditions in *A. thaliana* (Figure7). By contrast, however, the role of the transmembrane domain for targeting is less clear for ST (Table2). Whereas RRR and NRR are found in the *trans*-Golgi, NRN and RRN are seen in the ER and in the cytoplasm. The Golgi targeting of ST in plants might therefore require additional protein domains, like the stem region, or the chimeric NRN and RRN proteins might display aberrant features that are recognized by the ER quality control system.

**Table 2:** Summary of major findings

CTS-region	Subcellular location*	GnTI interaction (Co-IP)	GnTI interaction (FRET-FLIM)	*gntI* complementation
NNN	*cis/*medial*-*Golgi	+++	+++	+++
RRR	*trans-*Golgi	−	−	−
RNR	*cis*/medial-Golgi	−	−/+	++
NRR	*trans*-Golgi	−	−	−
RNN	*cis*/medial-Golgi	+++	+++	+++
NNR	*cis*/medial-Golgi	−	n/d	++
NRN	ER	n/a	n/a	n/a
RRN	ER	n/a	n/a	n/a

* Based on data from glycan-analyses as well as from quantification of confocal images.

n/a, not applicable; n/d, not done.

In the bilayer thickness model it was proposed that the length of the hydrophobic domain of glycosyltransferases could be implicated in sorting (Bretscher and Munro, 1993). This model is based on the finding that ER/Golgi-resident proteins tend to have a shorter transmembrane domain than plasma membrane proteins, and the observation that the bilayer length and composition is not homogenous throughout the endomembrane system. Hence, proteins with shorter hydrophobic stretches could be excluded from incorporation into thicker membrane regions, leading to a partitioning into different domains. For example, an increased plasma membrane expression of type–I protein chimeras was found when additional residues were inserted into the transmembrane domain. (Brandizzi *et al.*, 2002a). A seven amino acid increase of the transmembrane region of soybean Golgi-α-mannosidase I caused a shift from the *cis*/medial- to the *trans*-Golgi (Saint-Jore-Dupas *et al.*, 2006) indicating that the length of the hydrophobic stretch or the presentation of certain amino acids from the transmembrane domain could be essential factors for its sub-Golgi targeting. Our finding that the transmembrane domain is the major determinant for Golgi subcompartmentation of GnTI is consistent with such lipid-based sorting processes; however, the predicted length of the transmembrane spanning regions from *N. benthamiana* GnTI and ST are almost identical, which makes it unlikely that the number of amino acids alone contributes to the specific sub-Golgi concentration. Apart from the length of the transmembrane domain, we therefore propose that the amino acid composition could play a major role in the sub-Golgi localization of type–II membrane proteins. We and others have previously compared the length and the composition of the transmembrane domains of different Golgi-resident plant *N–*glycan processing enzymes and did not find any consensus sequence motif that distinguishes *cis*/medial- from *trans*-Golgi enzymes (Saint-Jore-Dupas *et al.*, 2006; van Dijk *et al.*, 2008; Schoberer and Strasser, 2011; Nikolovski *et al.*, 2012). The low number of plant type–II membrane proteins with confirmed sub-Golgi localization precludes a thorough comparison of sequence features; however, a more comprehensive bioinformatic approach could also not delineate a conserved sequence motif responsible for sub-Golgi targeting in a large number of mammalian proteins (Sharpe *et al.*, 2010). Whereas organelle-specific properties might discriminate between ER, Golgi and plasma membrane localization (Sharpe *et al.*, 2010; Nikolovski *et al.*, 2012), it is likely that Golgi subcompartmentation of individual proteins is determined by different intrinsic protein characteristics, rather than by a single mechanism.

In summary, we show in this study that sub-Golgi localization is crucial for the *in vivo* functionality of GnTI. Although it appears that the *cis*-/medial Golgi concentration of GnTI is mediated by the transmembrane domain, the homo- and heterodimer formation is strongly dependent on the stem region. Our data show that this protein–protein interaction is less important for sub-Golgi compartmentation of GnTI and its function *in vivo*; however, further studies are required to analyze the biological significance of the complexes and the interaction with other Golgi-resident proteins from the same or different biosynthetic pathways. Eventually, these studies will lead to a better understanding of mechanisms that govern protein homeostasis and function of glycan-modifying enzymes in the Golgi.

## Experimental procedures

### Cloning of constructs

The RNN expression construct was generated by ligation of two overlapping synthetic oligonucleotides (RSTC_1F/2R; Table S1) into *Xba*I/*Kpn*I-digested vector p20-GnTI-CTS-Fc-GFP (Schoberer *et al.*, 2009). The coding DNA sequences for all other chimeric CTS regions were obtained by custom DNA synthesis (GeneArt® Gene Synthesis). The DNA was excised by *Xba*I/*Bam*HI digestion and ligated into the *Xba*I/*Bam*HI sites of p20-Fc (expression of GFP_glyc_ tagged proteins), p31 (expression of mRFP tagged proteins), pF (expression of GALT fusions containing the catalytic domain of human β–1,4-galactosyltransferase), p57 (complementation of *gntI* plants with the full-length GnTI protein) or p46 (expression of proteins containing a CTS fused to the catalytic domain of *N. benthamiana* GnTI and GFP).

For the generation of pF vector, the human GALT catalytic domain was amplified by PCR using primers GALT18F/19R, and the *Bam*HI/*Xho*I-digested PCR product was ligated into the *Bam*HI/*Sal*I sites of pPT2M. For the generation of the pF-GCSI construct the CTS region from *A. thaliana* α–glucosidase I (Saint-Jore-Dupas *et al.*, 2006) was excised by *Xba*I/*Bam*HI digestion from GCSI-GFP_glyc_ and cloned into pF. In p31, p20-Fc and pF the expression is under the control of the CaMV *35S* promoter. Vector p57 was generated by the insertion of an assembled DNA fragment containing the 386–bp minimal promoter region from *A. thaliana GnTI*, the *A. thaliana GnTI* CTS region (AtNNN) and the *GnTI* catalytic domain into the *Hind*III/*Bam*HI site of vector p27GFP. For this purpose, the *GnTI* promoter region was amplified by PCR from genomic DNA using primers AthGnT_12F/13R, the CTS region was amplified with primers AthGnT_14F/16R and the catalytic domain was amplified using primers AthGnT_15F/9R. These DNA fragments were assembled using the Gibson Assembly Cloning Kit (NEB, http://www.neb.com). For vector p46 the catalytic domain of *N. tabacum* GnTI was amplified by PCR from p20-GnTI (Schoberer *et al.*, 2013) using primers NtGnTI_19F/31R. The PCR product was *Bam*HI/*Bgl*II–digested and cloned into *Bam*HI digested vector p46. In p46, the expression of proteins is under the control of the *A. thaliana* ubiquitin 10 promoter. The construct for expression of the mAb and for the expression of RRR-mRFP (ST-mRFP), RRR-GFP (ST-GFP), NNN-GFPglyc (GnTI-CTS-GFP_glyc_), NNN-mRFP (GnTI-CTS-mRFP), MNS1-GFP_glyc_, GCSI-GFP_glyc_, GnTI-mRFP, MNS1-mRFP and AtCASP-mRFP were all available from previous studies (Strasser *et al.*, 2009; Schoberer *et al.*, 2010, 2013).

### LC-ESI-MS analysis

Five-week-old *N. benthamiana* plants were used for *Agrobacterium tumefaciens*-mediated transient expression of indicated constructs using the agroinfiltration technique, as described previously (Schoberer *et al.*, 2009). Expressed CTS-GFP_glyc_ chimera or the mAb were purified 48 h after infiltration. A total of 1 g of infiltrated leaves was harvested, homogenized in liquid nitrogen using a mixer mill and resuspended in 600 μL of pre-cooled extraction buffer (1× PBS). After a brief incubation on ice, the extract was cleared by centrifugation (9000 ***g*** for 20 min at 4°C) and incubated for 1.5 h at 4°C with 20 μL of rProteinA Sepharose™ Fast Flow (GE Healthcare, http://www.gehealthcare.com). The sepharose was collected by centrifugation, washed three times with 1× PBS using Micro Bio-Spin™ Chromatography Columns (Bio-Rad, http://www.bio-rad.com) and the bound protein was eluted by incubation in Laemmli buffer for 5 min at 95°C. Approximately 1 μg of chimeric CTS-GFP_glyc_ or mAb was separated by SDS–PAGE (10%) under reducing conditions and stained with Coomassie Brilliant Blue. The corresponding protein band was excised from the gel, destained, carbamidomethylated, in-gel trypsin digested and analyzed by liquid chromatography electrospray ionization mass spectrometry (LC-ESI-MS), as described in detail previously (Stadlmann *et al.*, 2008; Schoberer *et al.*, 2009). A detailed explanation of *N–*glycan abbreviations can be found at http://www.proglycan.com.

### Complementation of *A. thaliana gntI* plants

*Arabidopsis thaliana gntI* knock-out plants (SALK_073560) (Kang *et al.*, 2008) were transformed with different p57 constructs by floral dipping, as described previously (Strasser *et al.*, 2004). Hygromycin-resistant plants were screened by PCR with GnTI and ST-specific primers, and selected PCR products were subjected to DNA sequencing. Proteins were extracted from leaves of 5–week-old plants, subjected to SDS-PAGE (10%) under reducing conditions and analyzed by immunoblotting with anti-horseradish peroxidase antibodies (anti-HRP; Sigma-Aldrich, http://www.sigmaaldrich.com) that bind to complex *N–*glycans carrying β–1,2-xylose and core α–1,3-fucose residues (Strasser *et al.*, 2004).

### Confocal imaging of fluorescent protein fusions

Leaves of 5–week-old *N. benthamiana* plants were infiltrated with agrobacterium suspensions carrying the protein(s) of interest with the following optical densities (OD_600_): NNN, RRR, NNR, RRN, NRN, RNR, RNN and NRR, 0.05; mRFP-AtCASP, 0.10; RRR-mRFP, 0.07. High-resolution images were acquired 2 and 3 days post infiltration (dpi) on an upright Leica SP5 II confocal microscope using the Leica las af software system (http://www.leica.com). GFP and mRFP were excited with 488- and 561–nm laser lines, respectively, and detected at 500–530 and 600–630 nm, respectively. Dual-color image acquisition of cells expressing both GFP and mRFP was performed simultaneously. Post-acquisition image processing was performed in Adobe photoshop cs5.

### Co-localization analyses of co-expressed fluorescent protein fusions

Images of cells expressing the GFP_glyc_-fused protein of interest together with the *cis*/medial-Golgi marker mRFP-AtCASP (Renna *et al.*, 2005; Osterrieder *et al.*, 2009) and the non-plant *trans*-Golgi marker RRR-mRFP (Boevink *et al.*, 1998; Renna *et al.*, 2005), respectively, were acquired 3 dpi without Golgi stack immobilization under non-saturating conditions using zoom factor 5 and a 63 × /1.40 NA oil immersion objective for NNN, RRR, and NRR, and using zoom factor 6 and a 40 × /1.25 NA oil immersion objective for NNR, RNN, and RNR. The pinhole was set to 1 airy unit, and background noise was reduced by line averaging of 8. Only cells with comparable GFP and mRFP fluorescence levels were considered for analysis. Side-on views of dual-labeled Golgi stacks were recorded preferentially, as the degree of overlap between two colors appeared clearer. The images obtained were used for co-localization analysis using the Pearson's correlation coefficient. Calculations were made on 28–38 confocal images per co-expressed combination using imagej 1.46 m plug-in jacop (Bolte and Cordelières, 2006). As every image contained between five and 10 Golgi bodies, 140–380 Golgi bodies were analyzed for each combination. For a graphical display of the distribution of GFP and mRFP fluorescence intensities across stacks, fluorescence intensity profiles (*x*–axis, length in μm; *y*–axis, normalized intensity) were generated by drawing a line across dual-labelled Golgi stacks using the ‘Line Profile’ intensity tool of the Leica las af software. Statistical analyses were performed using the Student's *t*–test for the comparison of two samples, assuming equal variances (Figure S3).

### FRET-FLIM data acquisition and analysis

Infiltrated leaf samples were excised and, prior to image acquisition, treated for 45–60 min with the actin-depolymerizing agent latrunculin B (stock solution at 1 mm in dimethyl sulphoxide; Calbiochem, now EMD Millipore, https://www.emdmillipore.com) at a concentration of 25 μm to inhibit Golgi movement (Brandizzi *et al.*, 2002b). 2P-FRET-FLIM data capture was performed as described previously (Sparkes *et al.*, 2010; Schoberer *et al.*, 2013) using a two-photon excitation microscope at the Central Laser Facility of the Rutherford Appleton Laboratory. Briefly, a two-photon microscope was constructed around a Nikon TE2000–U inverted microscope using custom-made XY galvanometers (GSI Lumonics, http://www.gsig.com). Laser light at a wavelength of 920 ± 5 nm was obtained from a mode-locked titanium sapphire laser (Mira 900F; Coherent Lasers, http://www.coherent.com), producing 180–fs pulses at 75 MHz. Two-photon excitation at 920 nm was chosen to allow reduced autofluorescence emission from chloroplast and guard cells. The laser beam was focused to a diffraction-limited spot through a VC 60 × /1.2 NA water immersion objective (Nikon, http://www.nikon.com). Fluorescence emission was collected without descanning, bypassing the scanning system, and passed through a BG39 (Comar, http://www.comaroptics.com) filter to block the near infrared laser light. Line, frame, and pixel clock signals were generated and synchronized with an external fast microchannel plate photomultiplier tube (MCP-PMT; Hamamatsu R3809U, http://www.hamamatsu.com) used as the detector. These were linked via a time-correlated single-photon-counting PC module SPC830 (Becker and Hickl, http://www.becker-hickl.com) to generate the raw FLIM data. Prior to FLIM data collection, the GFP and mRFP expression levels in the plant specimens within the region of interest were confirmed using a Nikon eC1 confocal microscope with excitation at 488 and 543 nm, respectively. A 633–nm interference filter was used to further minimize the contaminating effect of chlorophyll autofluorescence emission, which would otherwise obscure the mRFP emission. FLIM images were analyzed by obtaining excited-state lifetime values of a single cell. Calculations and image processing was performed using SPCIMAGE (Becker and Hickl). Lifetime values were collected on a single pixel basis from the center of individual Golgi bodies. Decay curves of a single point highlight an optimal single exponential fit when chi-square (χ^2^) values are 1 (points with χ^2^ from 0.9 to 1.4 were taken). The collected data values were used to generate histograms depicting the distribution of lifetime values of all data points within the samples. Results are from two or three independent experiments (>150 Golgi stacks from 12–13 cells in total).

An observed protein–protein interaction is described by the decrease of the donor fluorescence lifetime (quenching) as a result of energy transfer to the acceptor (Gadella and Jovin, 1995; Krishnan *et al.*, 2003), which can be calculated by measuring the fluorescence lifetime of the donor in the presence and absence of the acceptor (Bastiaens and Squire, 1999), and can be expressed as a percentage of the donor lifetime, a value referred to as ‘energy transfer efficiency’ (*E*). The percentage efficiency (*E*%) can be calculated using equation 1. (1)
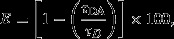
where τ_DA_ and τ_*D*_ are the mean pixel-by-pixel excited-state lifetimes of the donor in the presence and absence of the acceptor determined for each pixel. We have previously shown that a reduction of as little as ∼200 ps or 8% in the excited state lifetime of the GFP-labelled protein represents quenching through a protein–protein interaction (Stubbs *et al.*, 2005; Osterrieder *et al.*, 2009; Sparkes *et al.*, 2010; Schoberer *et al.*, 2013; Yadav *et al.*, 2013). As the instrument response (IR) in our set–up is determined to be less than 60 ps, there was no need to deconvolute the IR function from the sample data decay curves. Thus, lifetime differences of larger than 100 ps can be easily resolved.

### Co-purification and immunoblotting

Co-purification experiments were performed as previously described (Hüttner *et al.*. 2012). Briefly, leaves of 5–week-old *N. benthamiana* plants were co-infiltrated with agrobacteria (OD_600_ 0.2), containing p20-GnTI-CTS-Fc-GFP (NNN-GFP_glyc_) and different p31 constructs expressing the chimeric CTS regions (RNR, NRR, RNN and NNR) or controls (NNN and RRR) fused to mRFP. NNN-GFP_glyc_ was purified by binding to rProtein A–Sepharose™ Fast Flow, as described in detail by Hüttner *et al.* (2012), and immunoblot detection was performed using anti-GFP (MACS Miltenyi Biotec, http://www.miltenyibiotec.com) and anti-mRFP (ChromoTek, http://www.chromotek.com) antibodies. Similarly, constructs for expression of chimeric CTS-GFP_glyc_ were co-expressed with NNN-mRFP and analyzed in the same way. For analysis of MNS1-CTS and NNN interaction, MNS1-CTS-GFP_glyc_ was co-expressed with constructs expressing chimeric CTS-regions fused to mRFP, and for monitoring of the interaction between full-length proteins NNN-GNTI-GFP or RNR-GNTI-GFP (in p46) were co-expressed with NNN-GNTI-mRFP (in p31), co-purified using GFP-Trap-A beads (ChromoTek) and analyzed by immunoblotting.

## Supporting Information

Figure S1 LC-ESI-MS spectra of NRN and RRN glycoreporter fusion proteins also display the unglycosylated peptide.

Figure S2 Schematic presentation of the *N–*glycan processing pathway and the inhibition of processing by GALT action.

Figure S3 Statistical analyses of co-localization data.

Table S1 Primers used in this study.
