# Supplementary material for: The transmembrane domain of N –acetylglucosaminyltransferase I is the key determinant for its Golgi subcompartmentation
Source: Plant J. 2014 Sep 17;80(5):809–22. doi: 10.1111/tpj.12671 (PMC4282539; doi:10.1111/tpj.12671)
Supplement: Figure S1 — LC-ESI-MS spectra of NRN and RRN glycoreporter fusion proteins also display the unglycosylated peptide. [file tpj0080-0809-SD1.pdf]

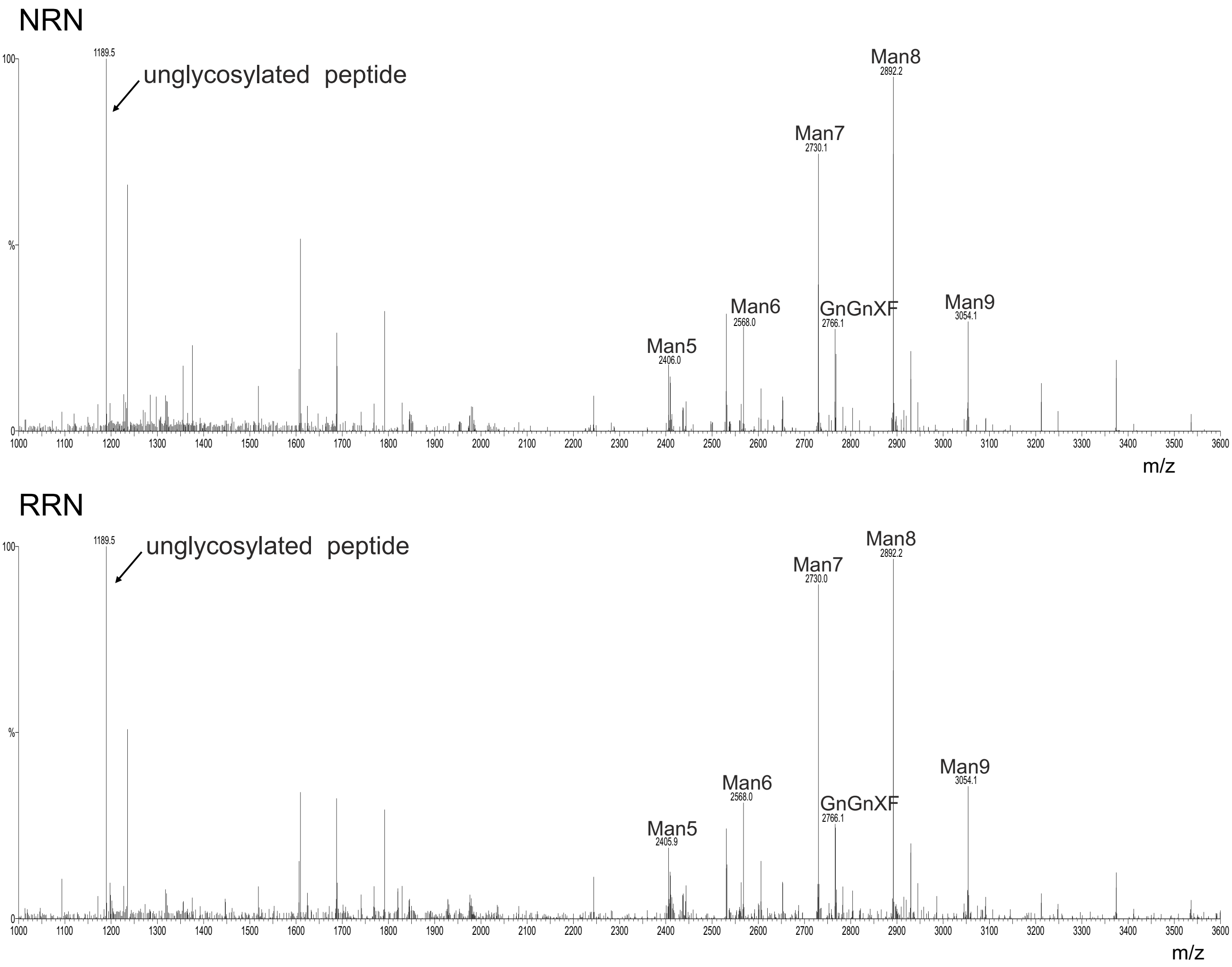

Figure S1: LC-ESI-MS spectra of NRN and RRN glycoreporter fusion proteins display also the unglycosylated peptide.

Mass spectra of glycopeptide 1 (EEQYNSTYR) derived from the glycoprotein part of GFP_{glyc} are shown. Man5 ($Man_5GlcNAc_2$) to Man9 ($Man_9GlcNAc_2$), oligomannosidic N-glycans, indicative of ER retention; GnGnXF ($GlcNAc_2XylFucMan_3GlcNAc_2$) complex N-glycan, processed in the Golgi apparatus. The peak corresponding to the unglycosylated peptide is indicated.
