# Supplementary material for: The transmembrane domain of N –acetylglucosaminyltransferase I is the key determinant for its Golgi subcompartmentation
Source: Plant J. 2014 Sep 17;80(5):809–22. doi: 10.1111/tpj.12671 (PMC4282539; doi:10.1111/tpj.12671)
Supplement: Figure S2 — Schematic presentation of the N–glycan processing pathway and the inhibition of processing by GALT action. [file tpj0080-0809-SD2.pdf]

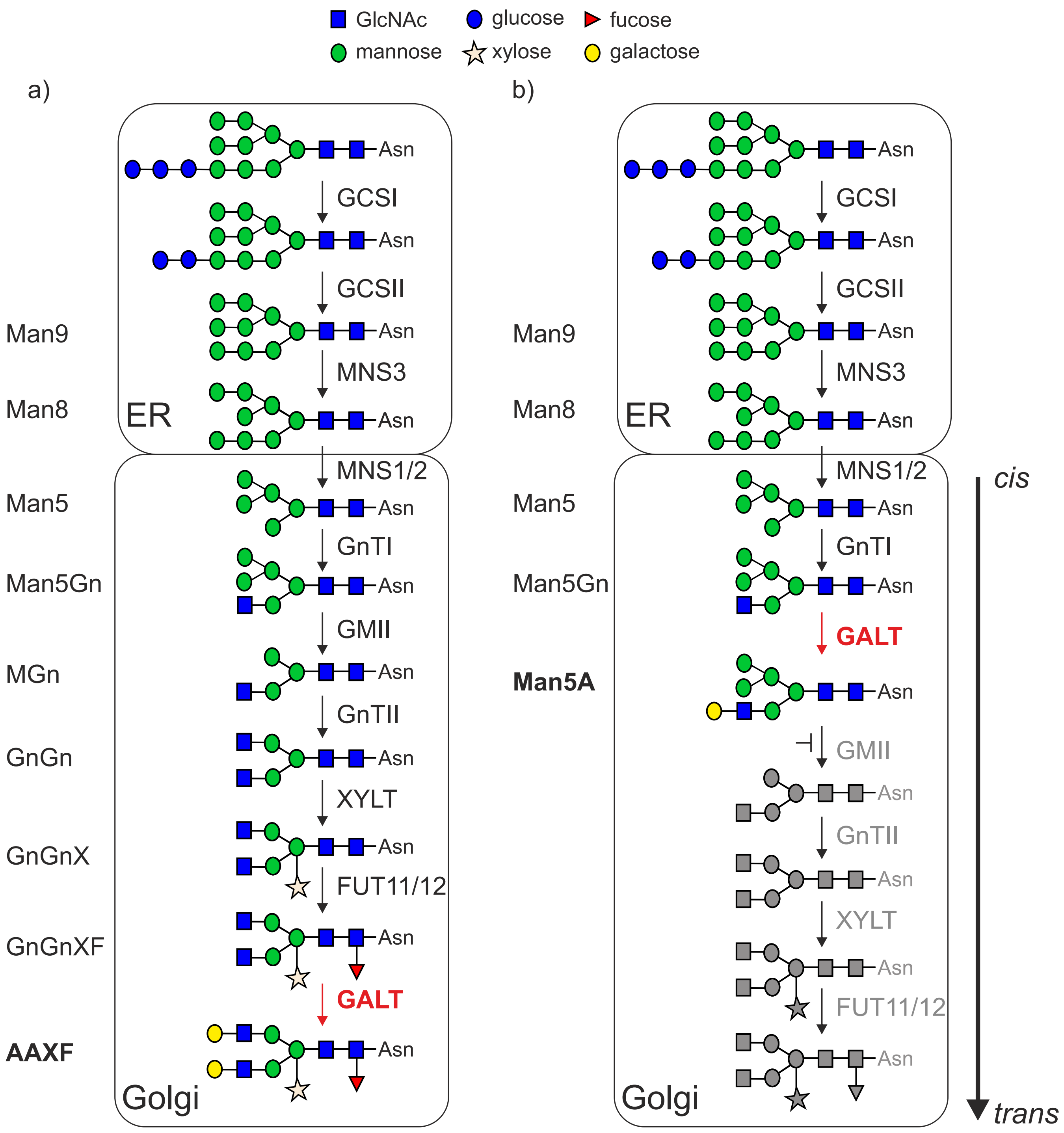

Figure S2: Schematic presentation of the N-glycan processing pathway and the inhibition of processing by GALT action at different sites within the Golgi.

N-glycan processing enzymes: GCSI: α-glucosidase I; GCSII: α-glucosidase II; MNS3: ER-α-mannosidase I; MNS1/2: Golgi-α-mannosidase I; GnTI: N-acetylglucosaminyltransferase I; GMII: Golgi-α-mannosidase II; GnTII: N-acetylglucosaminyltransferase II; XYLT: β1,2-xylosyltransferase; FUT11/FUT12: core α1,3-fucosyltransferase; GALT: human β1,4-galactosyltransferase.

a) GALT is targeted to the *trans*-Golgi (e.g. by fusion to RRR) and adds terminal galactose residues to the fully processed complex N-glycan GnGXF resulting in the formation of AAXF structures.

b) GALT is targeted to the *cis*/medial-Golgi (e.g. by fusion to NNN) and adds a single galactose residue to hybrid (Man5Gn) or other incompletely processed N-glycans. The presence of galactose (Man5A) inhibits N-glycan processing enzymes acting downstream of GnTI (structures depicted in grey).
