# Supplementary material for: The transmembrane domain of N –acetylglucosaminyltransferase I is the key determinant for its Golgi subcompartmentation
Source: Plant J. 2014 Sep 17;80(5):809–22. doi: 10.1111/tpj.12671 (PMC4282539; doi:10.1111/tpj.12671)
Supplement: Figure S3 — Statistical analyses of co-localization data. [file tpj0080-0809-SD3.pdf]

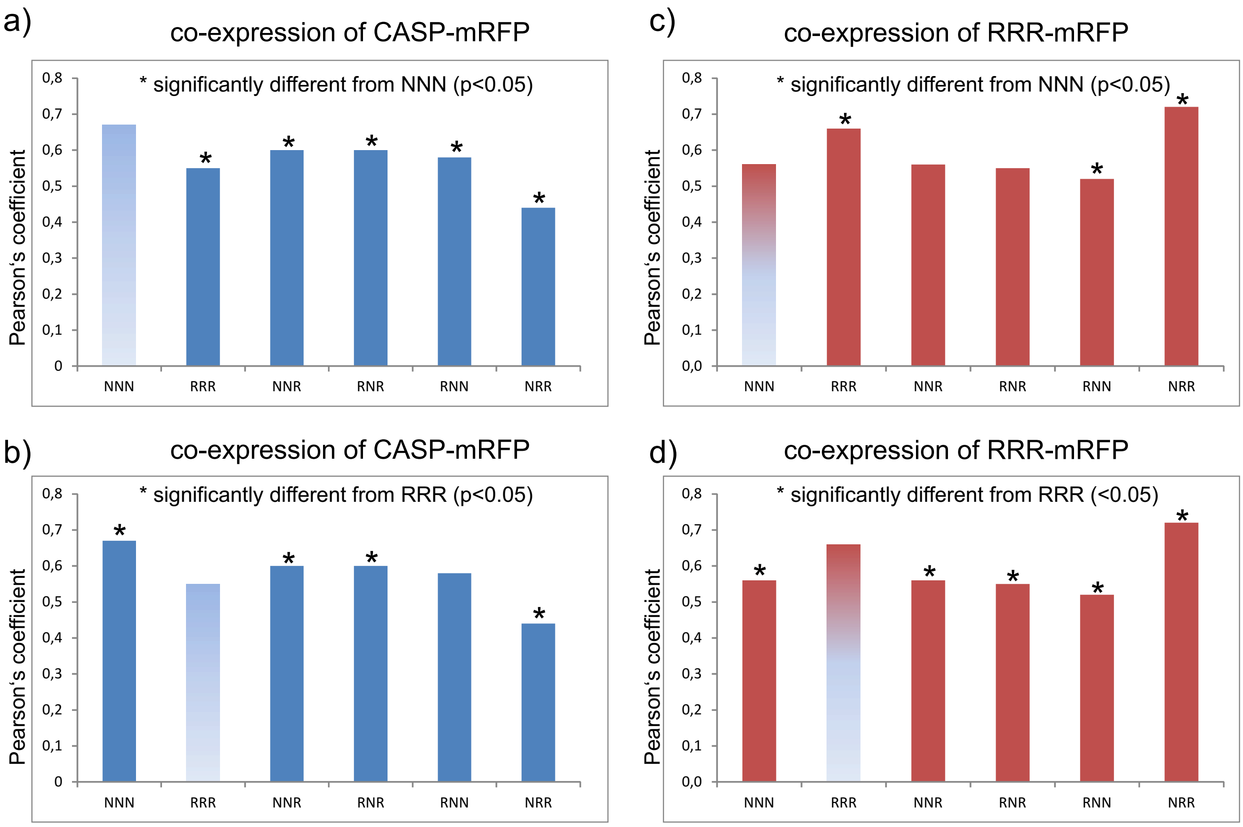

e)

Pearson's r	coexpression with CASP-RFP		
CTS	n	Mean	SD
NNN	38	0,67	0,06
RRR	29	0,55	0,08
NNR	37	0,60	0,08
RNR	34	0,60	0,06
RNN	34	0,58	0,07
NRR	36	0,44	0,11

n: number of confocal images used for quantification, each image contained 5-10 Golgi bodies

f)

Pearson's r	coexpression with RRR-mRFP		
CTS	n	Mean	SD
NNN	38	0,56	0,07
RRR	28	0,66	0,10
NNR	30	0,56	0,04
RNR	33	0,55	0,08
RNN	31	0,52	0,05
NRR	34	0,72	0,06

n: number of confocal images used for quantification, each image contained 5-10 Golgi bodies

Figure S3: Statistical analyses of co-localization data.
a) to d) Histograms of Pearson's correlation coefficient (r) (as shown in Figure 5b). Significant differences are marked by asterisks ($p<0.05$; two tailed t-Test: two-sample assuming equal variances).
a) Co-localization of NNN-GFP with CASP-mRFP, co-localization of RRR-GFP with CASP-mRFP, co-localization of chimeric GFPs with CASP-mRFP. NNN-GFP (*cis*/medial) co-expressed with CASP-mRFP (*cis*/medial) is reference value for calculation of p-values.
b) Same as in a) but RRR-GFP (*trans*) co-expressed with CASP-mRFP (*cis*/medial) is reference value for calculation of p-values.
c) Co-localization of NNN-GFP with RRR-mRFP, co-localization of RRR-GFP with RRR-mRFP, co-localization of chimeric GFPs with RRR-mRFP. NNN-GFP (*cis*/medial) co-expressed with RRR-mRFP (*trans*) is reference value for calculation of p-values.
d) Same as in c) but RRR-GFP (*trans*) co-expressed with RRR-mRFP (*trans*) is reference value for calculation of p-values.
e) Table with number of individual image counts, mean values and standard deviations (SD) for Pearson's coefficients (as shown in Figure 5b and Figure S3a and b).
f) Table with number of individual image counts, mean values and standard deviations (SD) for Pearson's coefficients (as shown in Figure 5b and Figure S3c and d).
