# Supplementary material for: The transmembrane domain of N –acetylglucosaminyltransferase I is the key determinant for its Golgi subcompartmentation
Source: Plant J. 2014 Sep 17;80(5):809–22. doi: 10.1111/tpj.12671 (PMC4282539; doi:10.1111/tpj.12671)
Supplement: Table S1 — Primers used in this study. [file tpj0080-0809-SD4.pdf]

Table S1. List of all used primers

RSTC_1F	CTAGAATGATTCATACCAACTTGAAGAAAAAGTAC
RSTC_2R	TTTTTCTTCAAGTTGGTATGAATCATT
GALT18F	TATAGGATCCGCCATCGGGCAGTCCT
GALT19R	TATACTCGAGCTAGCTCGGTGTCCCGATGTCC
AthGnT_12F	TAAGCTTCTGGCCGGTGGAGGTATTTT
AthGnT_13R	CCTCGCCATATCTAGAACAAACAACCAGATCAAAACCG
AthGnT_14F	TTGTTCTAGATATGGCGAGGATCTCGTGTGAC
AthGnT_16R	CGTCGGATCCCTGGCGGTTCTTCATATCTTCG
AthGnT_15F	GAACCGCCAGGGATCCGACGAAGAACTTGTGCAGCTTA
AthGnT_9R	TATAAGATCTGGAATTTCGAATTCCAAGCTGC
NtGnTI_19F	TATAGGATCCGACCAGGAGTGCCGACAGTTAAGGG
NtGnTI_31R	TATAAGATCTAGTATCTTCATTTCCGAGTTGTT
