# Supplementary material for: The transmembrane domain of N –acetylglucosaminyltransferase I is the key determinant for its Golgi subcompartmentation
Source: Plant J. 2014 Sep 17;80(5):809–22. doi: 10.1111/tpj.12671 (PMC4282539; doi:10.1111/tpj.12671)
Supplement: Supplementary file 5 — supplementary [file tpj0080-0809-SD5.docx]

**Figure S1:** LC-ESI-MS spectra of NRN and RRN glycoreporter fusion proteins display also the unglycosylated peptide.

Mass spectra of glycopeptide 1 (EEQYNSTYR) derived from the glycoprotein part of GFP_glyc_ are shown. Man5 (Man_5_GlcNAc_2_) to Man9 (Man_9_GlcNAc_2_), oligomannosidic N-glycans, indicative of ER retention; GnGnXF (GlcNAc_2_XylFucMan_3_GlcNAc_2_) complex N-glycan, processed in the Golgi apparatus. The peak corresponding to the unglycosylated peptide is indicated.

**Figure S2:** Schematic presentation of the N-glycan processing pathway and the inhibition of processing by GALT action at different sites within the Golgi.

N-glycan processing enzymes: GCSI: α-glucosidase I; GCSII: α-glucosidase II; MNS3: ER-α-mannosidase I; MNS1/2: Golgi-α-mannosidase I; GnTI: N-acetylglucosaminyltransferase I; GMII: Golgi-α-mannosidase II; GnTII: N-acetylglucosaminyltransferase II; XYLT: β1,2-xylosyltransferase; FUT11/FUT12: core α1,3-fucosyltransferase; GALT: human β1,4-galactosyltransferase.

a) GALT is targeted to the *trans*-Golgi (e.g. by fusion to RRR) and adds terminal galactose residues to the fully processed complex N-glycan GnGXF resulting in the formation of AAXF structures.

b) GALT is targeted to the *cis*/medial-Golgi (e.g. by fusion to NNN) and adds a single galactose residue to hybrid (Man5Gn) or other incompletely processed N-glycans. The presence of galactose (Man5A) inhibits N-glycan processing enzymes acting downstream of GnTI (structures depicted in grey).

**Figure S3:** Statistical analyses of co-localization data.

a) to d) Histograms of Pearson’s correlation coefficient (r) (as shown in Figure 5b). Significant differences are marked by asterisks (p<0.05; two tailed t-Test: two-sample assuming equal variances).

a) Co-localization of NNN-GFP with CASP-mRFP, co-localization of RRR-GFP with CASP-mRFP, co-localization of chimeric GFPs with CASP-mRFP. NNN-GFP (*cis*/medial) co-expressed with CASP-mRFP (*cis*/medial) is reference value for calculation of p-values.

b) Same as in a) but RRR-GFP (*trans*) co-expressed with CASP-mRFP (*cis*/medial) is reference value for calculation of p-values.

c) Co-localization of NNN-GFP with RRR-mRFP, co-localization of RRR-GFP with RRR-mRFP, co-localization of chimeric GFPs with RRR-mRFP. NNN-GFP (*cis*/medial) co-expressed with RRR-mRFP (*trans*) is reference value for calculation of p-values.

d) Same as in c) but RRR-GFP (*trans*) co-expressed with RRR-mRFP (*trans*) is reference value for calculation of p-values.

e) Table with number of individual image counts, mean values and standard deviations (SD) for Pearson’s coefficients (as shown in Figure 5b and Figure S3a and b).

f) Table with number of individual image counts, mean values and standard deviations (SD) for Pearson’s coefficients (as shown in Figure 5b and Figure S3c and d).
